# Genetic Diversity of Human Rotavirus A Among Hospitalized Children Under-5 Years in Lebanon

**DOI:** 10.3389/fimmu.2020.00317

**Published:** 2020-02-26

**Authors:** Houda H. Harastani, Lina Reslan, Ahmad Sabra, Zainab Ali, Moza Hammadi, Soha Ghanem, Farah Hajar, Ghassan M. Matar, Ghassan S. Dbaibo, Hassan Zaraket

**Affiliations:** ^1^Faculty of Medicine, Center for Infectious Diseases Research, American University of Beirut, Beirut, Lebanon; ^2^Department of Pediatrics and Adolescent Medicine, Faculty of Medicine, American University of Beirut, Beirut, Lebanon; ^3^Department of Experimental Pathology, Immunology, and Microbiology, Faculty of Medicine, American University of Beirut, Beirut, Lebanon; ^4^Department of Biochemistry and Molecular Genetics, Faculty of Medicine, American University of Beirut, Beirut, Lebanon

**Keywords:** human rotavirus, capsid proteins, diversity, vaccine, breakthrough, Rotarix, RotaTeq

## Abstract

Human rotavirus remains a major cause of gastroenteritis worldwide despite the availability of effective vaccines. In this study, we investigated the genetic diversity of rotaviruses circulating in Lebanon. We genetically characterized the VP4 and VP7 genes encoding the outer capsid proteins of 132 rotavirus-associated gastroenteritis specimens, previously identified in hospitalized children (<5 years) from 2011 to 2013 in Lebanon. These included 43 vaccine-breakthrough specimens and the remainder were from non-vaccinated subjects. Phylogenetic analysis of VP4 and VP7 genes revealed distinct clustering compared to the vaccine strains, and several substitutions were identified in the antigenic epitopes of Lebanese specimens. No unique changes were identified in the breakthrough specimens compared to non-breakthroughs that could explain the occurrence of infection in vaccinated children. Further, we report the emergence of a rare P[8] OP354-like strain with a G9 VP7 in Lebanon, possessing high genetic variability in their VP4 compared to vaccine strains. Therefore, human rotavirus strains circulating in Lebanon and globally have accumulated numerous substitutions in their antigenic sites compared to those currently used in the licensed vaccines. The successful spread and continued genetic drift of these strains over time might undermine the effectiveness of the vaccines. The effect of such changes in the antigenic sites on vaccine efficacy remains to be assessed.

## Introduction

Rotavirus-associated gastroenteritis (RVGE) constitutes a major public health burden in both industrialized and developing countries (1). Rotavirus (RV) is the most common etiological agent of gastroenteritis in children <5 years of age, accounting for around 215,000 annual deaths (1–4). Group A rotaviruses (RVAs), members of the *Reoviridae* family, are non-enveloped triple-layered particles that encompass a genome of eleven double-stranded RNA (dsRNA) segments coding for six structural viral proteins (VPs 1-4, 6, and 7) and five or six non-structural proteins (NSPs 1-5/6). RVAs are classified into G (glycoprotein) and P (protease-sensitive) genotypes based on the genes coding for the two outer capsid proteins, VP7 and VP4, respectively (5). So far, 36 G and 51 P genotypes have been identified (https://rega.kuleuven.be/cev/viralmetagenomics/virus-classification/rcwg). Nonetheless, epidemiological studies show that at least six human RVA G genotypes (G1-G4, G9, and G12) and three P genotypes (P[4], P[6], and P[8]) predominate globally in human infections (6).

Velasquez et al. showed that natural RVA infections can protect against subsequent infections (7). These findings constituted the basis for RVA vaccination, which is currently the most effective preventive tool. As of 2009, the World Health Organization (WHO) has recommended the use of RVA vaccines for infants worldwide as part of routine national immunization programs (NIPs) to reduce its public health burden (2, 8).

Currently, two live-attenuated oral RVA vaccines, RotaTeq® and Rotarix®, are licensed by the Food and Drug Administration (FDA) and approved by the Advisory Committee on Immunization Practices (ACIP) for human use (9, 10). RotaTeq® is a pentavalent human-bovine re-assorted RVA vaccine containing the human VP7 (G1-G4) and VP4 (P[8]) genes representing the most commonly circulating human RVA genotypes. On the other hand, Rotarix® is a monovalent RVA vaccine consisting of a single human G1P[8] strain (11). Both vaccines elicit heterotypic immunity and demonstrated high efficacy (>85%) against severe RVGE episodes (12, 13). The implementation of RVA childhood vaccination programs has resulted in a substantial decrease in RVA-associated hospitalizations in both developing and developed countries (13, 14).

Rotavirus is continuously evolving (15). Therefore, it is essential to study how vaccine-derived immunity shapes the evolution and spread of the major RVA strains that are targeted by the vaccines. In Lebanon, Rotarix® was introduced in the private market in late 2006 and RotaTeq® was first made available in 2009, but they have yet to be included in the NIP. Ali et al. reported an 18.1% vaccination rate among children <5 years of age hospitalized for gastroenteritis in Lebanon (16). During the 30-month study period, RVA infections were confirmed in 30.3% of children younger than 5 years of age hospitalized for acute gastroenteritis (GE) (16). Vaccine effectiveness was estimated at 68.4%, consistent with studies from other developing countries (17). Nonetheless, the vaccine effectiveness was found to be higher for the G1P[8] compared to the other genotypes (16). Vaccine-breakthrough infections were detected in 18.8% of the vaccinated children (16). In the current study, we genetically characterized RVA specimens collected from vaccinated and non-vaccinated children and compared them with the vaccine and other globally circulating strains.

## Materials and Methods

### Sample Collection

The specimens were detected during a prospective, multicenter hospital-based RVA surveillance study that was conducted from January 2011 to June 2013. The study population included hospitalized children <5 years of age admitted with acute GE recruited at seven centers distributed over North, Central, and South Lebanon (16). Subjects were classified as unvaccinated if they did not receive any RVA vaccination, and as vaccinated if they received at least one dose of an RVA vaccine. Clinical severity of diarrheal episodes was assessed using the previously described 20 point Vesikari scoring system manual (18). A breakthrough infection was defined as a laboratory-confirmed RVA infection at least 14 days after the latest vaccination dose.

### RNA Extraction and Reverse Transcription-PCR (RT-PCR)

Viral RNA was extracted from 10% (w/v for solid and v/v for liquid) stool suspensions for all RVA enzyme immunoassay positive samples using a QIAamp Viral RNA Mini Kit (Qiagen, Germany) according to manufacturer's instructions. The extracted RNA was denatured at 97°C for 5 min and reverse transcription followed by polymerase chain reaction (RT-PCR) was carried out using the Qiagen OneStep RT-PCR Kit (Qiagen, Germany) with the following thermal cycling conditions: initial reverse transcription step at 42°C for 30 min, followed by initial denaturation at 95°C for 15 min, 35 cycles of amplification and a final extension at 72°C for 7 min. Amplification of the complete VP7 gene and partial VP4 gene (VP8^*^ region) was carried out using previously described primers (19, 20). The VP5^*^ region (945 bp) of the VP4 gene was amplified using primers designed specifically for this study to cover all epitopes present in the VP5^*^ region: VP5-F (5′-GGTGGAAGARTATGGACATTTCA-3′) and VP5-R (5′-CATAATTGGAGTCTGATAATCATC-3′).

### Sanger Sequencing and Phylogenetic Analysis

PCR amplicons were Sanger sequenced at Macrogen Inc. (Seoul, Republic of Korea). Nucleotide sequences were assembled using ClustalW tool included in BioEdit Sequence Alignment Editor v7.2.5. The corresponding G and P genotypes were assigned by RotaC v2.0 software (http://rotac.regatools.be/) for automated genotyping of RVA and confirmed using the Basic Local Alignment Search Tool (BLAST) available on GenBank database (http://blast.ncbi.nlm.nih.gov/). To determine the extent of intra- and inter-genotypic diversity among VP7 and VP4 genes, phylogenetic trees were constructed using the neighbor-joining (NJ) algorithm, and evolutionary distances were computed using the Maximum Composite Likelihood method. To further investigate the genetic relatedness of the isolates, VP7 and VP4 gene sequences were compared with sequences of Rotarix® and RotaTeq®, sequences of potential candidate RV vaccine strains, namely the human neonatal strains RV3 (21) and 116E (22), as well as regional and global strains. Tree topology was evaluated using bootstrap re-sampling of 1,000 pseudoreplicate data sets in the MEGA v10.1.7 analytical package. Nucleotide and amino acid pairwise calculations were also conducted in MEGA v10.1.7 using the P-distance method. RVA nomenclature was according to the Rotavirus Classification Working Group (RCWG) (23).

### Prediction of Glycosylation Sites

The N-linked glycosylation sites (N-X-S or T patterns, where x can be any amino acid other than proline) were predicted using the NetNGlyc 1.0 server (http://www.cbs.dtu.dk/services/NetNGlyc/).

### Ethics

The work was carried out according to the guidelines of Good Clinical Practice outlined in the 1996 version of the Declaration of Helsinki. The study was approved by the Institutional Review Board (IRB) of each participating center before initiating the study. Specimens were collected upon obtaining a signed consent from the legal guardian/parent approving participation in the study.

### Data Availability

Reference and potential candidate vaccine strains were retrieved from GenBank: Rotarix® (JN849114 and JN849113), RotaTeq® (GU565057, GU565068, GU565079, GU565090, and GU565044), RV3 (FJ998278) and 116E (L14072). VP4 and VP7 sequences were submitted to GenBank under accession numbers: VP4 (KR181886 to KR181927, KT257309 to KT257314, and MH591315 to MH591398) and VP7 sequences (KR094069 to KR094114, KR069121, KT257308, and MH591231 to MH591314).

## Results

Forty-three RVA specimens from breakthrough infections and 89 specimens from non-breakthrough infections were included in the study. Thirty-five (83%) of the breakthrough specimens were from subjects vaccinated with Rotarix®, seven (15%) with RotaTeq®, and one from an undocumented vaccine type. These strains included 8 G1P[8], 15 G2P[4], 1 G2P[8], 7 G4P[8], 11 G9P[8], and 1 G9P[4]. The average Vesikari score was reported to be 13.8±2.7 for the breakthrough cases (Table 1) compared to 14±2.6 for the non-breakthrough ones (data not shown).

**Table 1:** Demographics, RVA vaccination, and the clinical score of Lebanese breakthrough cases included in this study.

**Specimen ID**	**Gender**	**Date of admission (MM/YYYY)**	**Age (y)**	**Vaccine**	**No. of doses**	**Days to infection since last dose**	**Vesikari score**
LBN/A004/G1P[8]	M	02/2011	3.15	RV1	1	1086	14
LBN/H006/G1P[8]	F	05/2011	2.19	RV1	1	ND^3^	12
LBN/N222/G1P[8]	M	12/2011	0.68	RV1	1	30	13
LBN/H151/G1P[8]	M	12/2011	1.38	RV1	2	260	16
LBN/M154/G1P[8]	M	07/2011	3.87	RV5	3	ND	16
LBN/N280/G1P[8]	F	01/2012	0.42	RV1	1	73	18
LBN/N318/G1P[8]	F	05/2012	0.91	RV1	1	171	16
LBN/A170/G1P[8]	F	02/2013	1.69	RV1	2	513	11
LBN/A081/G2P[4]	F	11/2011	2.45	RV1	2	773	16
LBN/A082/G2P[8]	M	11/2011	2.18	RV1	2	417	12
LBN/A094/G2P[4]	F	12/2011	0.45	RV1	1	65	13
LBN/H159/G2P[4]	F	03/2012	2.73	RV5	3	ND	19
LBN/NG034/G2P[4]	F	01/2012	1.62	RV1	2	174	11
LBN/H178/G2P[4]	F	04/2012	0.71	RV1	1	ND	12
LBN/H195/G2P[4]	M	07/2012	0.56	RV1	1	ND	15
LBN/H198/G2P[4]	F	12/2012	1.8	RV1	1	ND	15
LBN/NG144/G2P[4]	M	11/2012	0.81	RV1	2	170	16
LBN/H237/G2P[4]	M	12/2012	1.47	RV1	2	524	15
LBN/H239/G2P[4]	M	12/2012	1.02	RV1	2	106	16
LBN/H248/G2P[4]	M	06/2013	2.99	ND^1^	ND^2^	ND	12
LBN/NG168/G2P[4]	M	01/2013	1.46	RV1	2	423	9
LBN/NG169/G2P[4]	F	01/2013	0.96	RV1	2	229	10
LBN/NG189/G2P[4]	F	02/2013	3.37	RV1	2	1185	12
LBN/NG198/G2P[4]	M	02/2013	0.9	RV1	2	263	11
LBN/M078/G4P[8]	M	05/2011	0.26	RV5	1	ND	11
LBN/NG064/G4P[8]	M	03/2012	0.79	RV1	2	344	15
LBN/NG068/G4P[8]	F	03/2012	1.23	RV1	2	295	13
LBN/R077/G4P[8]	F	04/2012	2.16	RV5	3	634	14
LBN/H250/G4P[8]	F	10/2013	1.75	RV5	1	ND	12
LBN/NG160/G4P[8]	M	01/2013	0.42	RV1	2	28	9
LBN/A164/G4P[8]	M	02/2013	1.17	RV1	2	342	12
LBN/M006/G9P[8]	F	08/2011	1.57	RV1	1	328	19
LBN/N486/G9P[8]	F	12/2012	4.24	RV1	2	1058	18
LBN/N488/G9P[8]	F	12/2012	0.95	RV1	2	49	11
LBN/A014/G9P[8]	M	03/2011	2.1	RV1	2	658	16
LBN/N394/G9P[8]	M	08/2012	0.9	RV1	2	140	17
LBN/M036/G9P[8]	F	03/2011	1.02	RV5	3	ND	18
LBN/N087/G9P[8]	F	08/2011	2.59	RV1	2	427	12
LBN/NG142/G9P[8]	F	11/2012	0.47	RV1	2	109	9
LBN/NG164/G9P[8]	M	01/2013	1.04	RV1	2	106	13
LBN/M199/G9P[8]	F	03/2013	0.24	RV1	1	26	15
LBN/NG203/G9P[8]	F	03/2013	0.25	RV1	2	31	11
LBN/H017/G9P[4]	M	01/2011	0.55	RV5	1	301	18

1 *ND, not-determined; clinical records reveals that the subject received RVA vaccination, however, unnoted whether it was a Rotarix® or a RotaTeq®*.

2 *Number of doses of administered RVA vaccination was not determined in clinical records*.

3 *Days to infection since last dose was undetermined; however, it is confirmed that latest RVA vaccination dose was administered at least >1 month prior to infection*.

### Nucleotide Sequence and Phylogenetic Analysis of VP7 and VP4 Genes

**Figure 1:**
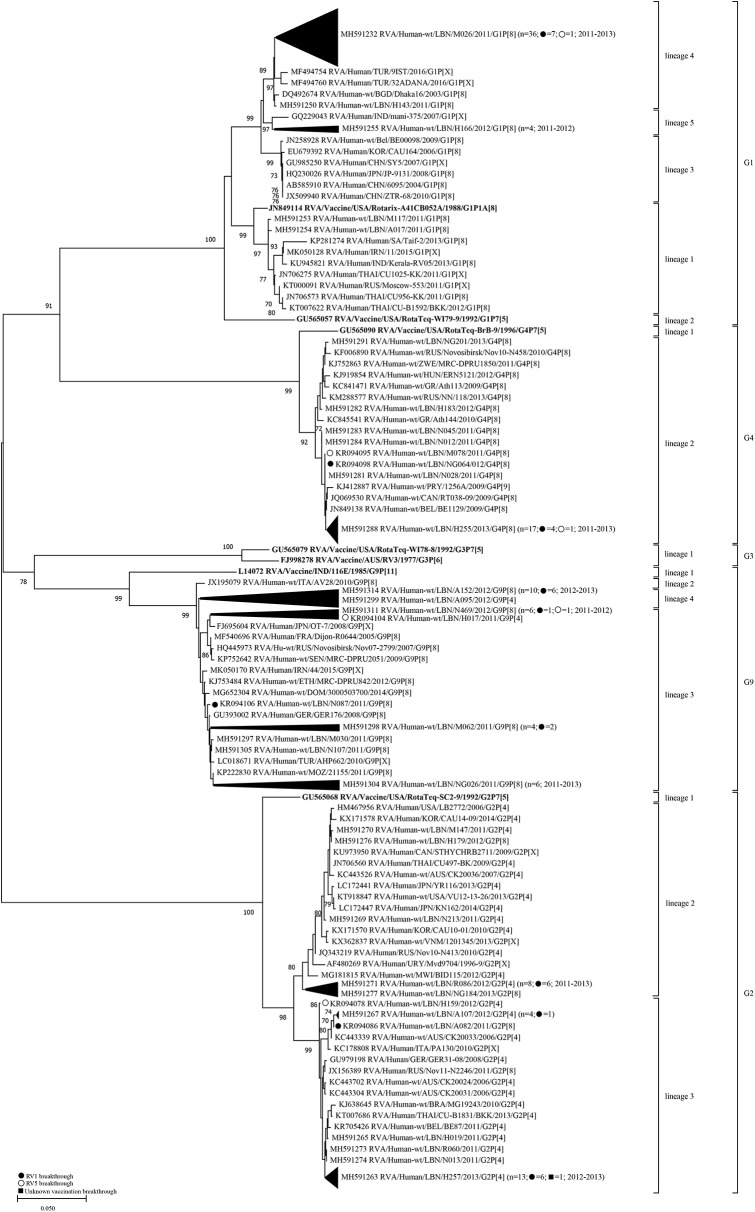
Phylogenetic dendrogram of VP7 genes (reading frame sequences 1-951) of RVA strains circulating in Lebanon (2011-2013) in relation to globally representative strains and those of Rotarix® and RotaTeq®. Phylogenetic analysis was performed using the neighbor-joining method. Horizontal branch lengths are drawn to scale (nucleotide substitutions per base) and bootstrap values (1000 pseudo-replicates) are shown at branch nodes. The annotation indicates strain ID/genotype/year of isolation. Branches comprised solely of Lebanese strains were collapsed and one representative strain was indicated for each branch. The total number of specimens in each collapsed branch and the years of isolation are indicated in parenthesis. The number of breakthrough specimens for each collapsed branch is also shown.

Based on the phylogenetic tree analysis of the VP7 gene, we arbitrary designated five lineages (1–5) within the G1-genotype. The Lebanese strains belonged to lineages 1, 4, and 5; whereas, Rotarix® and RotaTeq® G1 sequences belonged to lineages 1 and 2, respectively. Two G1 non-breakthrough specimens from 2011 clustered in lineage 1 along with the G1 of Rotarix® and other global strains, while the remaining specimens belonged to non-vaccine lineages (Figure 1). Lineage 1 included G1P[8] strains from several countries including Saudi Arabia, India, Iran, and Thailand. All of the breakthrough viruses and the majority of the non-breakthrough specimens collected between 2011 and 2013 belonged to lineage 4 clustering with strains from Tukey and Bangladesh with 98.5–99.6% nucleotide similarity. G1-lineage 5 was formed of only non-breakthrough specimens (*n* = 4) from 2011 to 2012, which clustered with an Indian strain (mani-375) with 98.3–98.7% nucleotide similarity. Lebanese G1 RVA specimens exhibited higher nucleotide (93.9–97.9%) and amino acid similarities (94.3–97.8%) to G1 strain of Rotarix® than that included in RotaTeq® (91.1–93.0% nucleotide and 93.1–95.3% amino acid identities) (Table 2).

**Table 2:** Distance matrix for VP7 and VP4 based on nucleotide and amino acid similarities.

**Nucleotide identity % (amino acid identity %)^a^**								
		**G1-Rotarix^®^**	**G1-RotaTeq^®^**	**G2-RotaTeq^®^**	**G3-RotaTeq^®^**	**G4-RotaTeq^®^**	**G3-RV3**	**G9-116E**
VP7	Breakthrough G1	94.1–94.4 (94.6–95.0)	91.7–92.0 (93.7–94.0)	74.3–74.6 (76.0)	75.1–75.2 (80.1–80.4)	76.2–76.8 (76.0–76.3)	74.6–74.8 (80.8–81.1)	75.7–76.0 (77.6–77.9)
	Non-breakthrough G1	93.9–97.9 (94.3–97.8)	91.1–93.0 (93.1–95.3)	73.7–74.8 (75.1–76.3)	74.9–75.4 (79.2–80.8)	75.8–77.0 (76.0–76.7)	74.3–75.1 (79.8–81.4)	75.4–76.0 (77.3–78.5)
	Breakthrough G2	72.9–73.8 (74.4–75.4)	73.0–73.8 (75.1–76.0)	93.5–94.3 (94.6–95.6)	73.7–74.0 (74.4–75.1)	72.3–72.9 (70.7–71.6)	73.3–73.8 (75.1–76.0)	74.2–74.7 (74.1–75.4)
	Non-breakthrough G2	72.8–73.8 (74.4–75.1)	72.9–73.7 (74.8–75.7)	93.5–93.8 (95.0–95.6)	73.5–74.1 (74.1–75.1)	72.2–73.0 (71.0–71.6)	73.0–73.9 (74.8–76.0)	73.9–74.7 (74.4–75.4)
	Breakthrough G4	75.8–76.2 (76.7–77.4)	75.2–75.6 (75.5–76.1)	71.7–72.1 (72.3–73.0)	74.2–74.5 (74.8–75.2)	94.8–95.2 (95.3–96.2)	74.7–74.9 (75.2–75.5)	74.3–74.6 (77.0–77.8)
	Non-breakthrough G4	75.8–76.3 (76.7–77.4)	75.2–75.6 (75.5–76.1)	71.7–72.3 (72.3–73.0)	74.2–74.5 (75.2)	94.8–95.3 (95.3–96.5)	74.7–75.2 (75.5)	74.4–74.7 (77.0–77.7)
	Breakthrough G9	75.8–76.8 (81.1–81.7)	74.8–76.0 (80.4–81.1)	75.2–75.7 (78.9–79.2)	79.3–79.6 (86.1–86.4)	74.4–75.2 (79.2–79.8)	78.1–78.4 (86.8–87.4)	88.2–88.9 (91.5–92.4)
	Non-breakthrough G9	75.6–76.7 (80.8–81.4)	74.6–76.0 (80.1–81.1)	74.9–75.6 (78.5–79.2)	79.2–79.8 (85.8–86.4)	74.4–75.5 (79.2–79.8)	78.1–78.8 (86.8–87.4)	87.8–88.5 (91.5–92.4)
		**P[8]-Rotarix**^®^	**P[8]-RotaTeq**^®^					
VP4	Breakthrough P[8]	88.6–90.7 (90.0–94.3)	88.8–93.5 (90.8–95.4)					
	Non-breakthrough P[8]	88.4–91.2 (90.0–94.8)	88.4–93.5 (90.6–95.9)					
	Breakthrough P[4]	85.5–86.3 (87.3–87.8)	85.7–86.1 (88.6–89.5)					
	Non-breakthrough P[4]	85.8–86.3 (87.3–88.0)	85.7–86.1 (88.9–89.7)					

a *Percent nucleotide and amino acid identities of VP4 (reading frame sequences 49–1422) and VP7 (reading frame sequences 1–951) genes of the Lebanese RVA strains were compared to Rotarix® and RotaTeq® vaccine strains as well as to potential neonatal RV vaccine strains*.

Lebanese G2 RVA specimens clustered in two lineages (2 and 3) that were distinct from G2 of RotaTeq® (lineage 1). Strains in G2-lineage 2 (*n* = 12) clustered with strains from Australia, Canada, USA, Russia, Uruguay, Malawi, and Far East countries with 95.8–99.9% nucleotide identity. Strains in G2-lineage 3 (*n* = 22) clustered with strains from Europe, Thailand, and Australia with 96.0–99.4% nucleotide similarity. Collectively, the Lebanese G2 RVA specimens showed 93.5–94.3% nucleotide and 94.6–95.6% amino acid identities with the G2 strain of RotaTeq®.

Lebanese G4 RVA specimens co-clustered in G4-lineage 2 with specimens from Europe, Canada, and Zimbabwe with 98.4–99.7% nucleotide similarity. Lebanese G4 RVA shared 94.8–95.3% nucleotide and 95.3–96.5% amino acid identities with the G4 strain of RotaTeq® (G4-lineage 1).

Lebanese G9 RVA belonged to two lineages: G9-lineage 3 (*n* = 20) and G9-lineage 4 (*n* = 11) clustering with RVA strains from Europe, Ethiopia, Iran, and Japan with 98.4–99.5% nucleotide similarity. The G9 RVA specimens had 87.8–88.9% nucleotide and 91.5–92.4% amino acid similarities with the vaccine candidate strain G9-116E (22).

Aside from the G1-lineage 4 specimens, the Lebanese vaccine breakthrough and non-breakthrough specimens co-clustered together in the lineages mentioned above suggesting high similarity among these strains.

**Figure 2:**
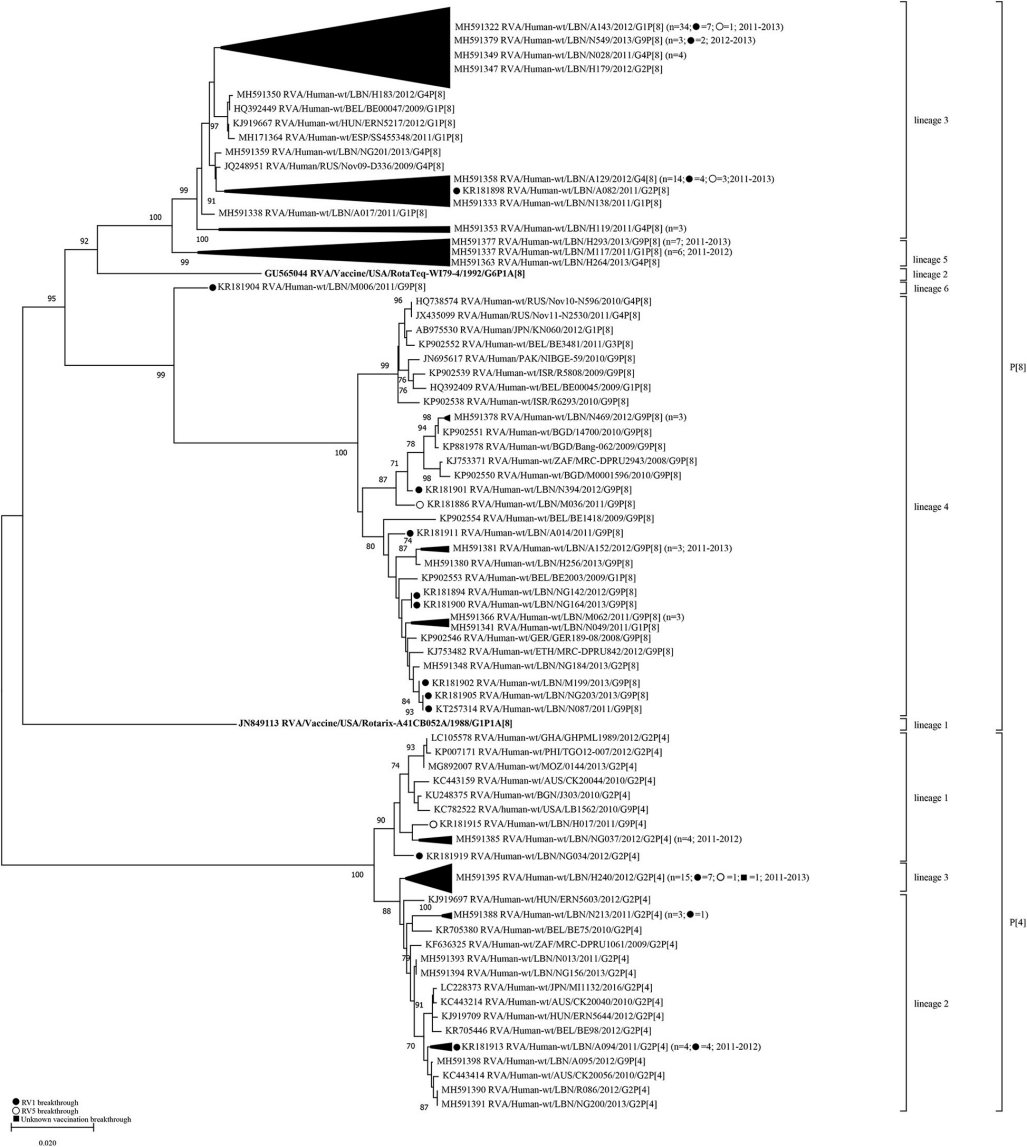
Phylogenetic dendrogram of VP4 genes (reading frame 49-1422) of RVA strains circulating in Lebanon (2011-2013) in relationship globally representative strains and those of Rotarix® and RotaTeq®. Phylogenetic analysis was performed using the neighbor-joining method. All horizontal branch lengths are drawn to scale (nucleotide substitutions per base) and bootstrap values (1000 pseudo-replicates) are shown at branch nodes. The annotation indicates strain ID/genotype/year of isolation. Branches comprised solely of Lebanese strains were collapsed and one representative strain was indicated for each branch. The total number of specimens in each collapsed branch and the years of isolation are indicated in parenthesis. The number of breakthrough specimens for each collapsed branch is also shown.

Similar to VP7, analysis of the VP4 gene segment showed that Lebanese RVA specimens formed distinct clusters compared to the vaccine strains (Figure 2). Six lineages (1–6) could be designated within the P[8] genotype. The P[8] of Rotarix® and RotaTeq®, clustered in lineages 1 and 2, respectively. The majority of Lebanese P[8] RVA specimens (*n* = 64) belonged to lineage 3 clustering with strains from Europe with 88.6–99.8% nucleotide similarity. Lebanese strains belonging to P[8]-lineage 4 (*n* = 20) clustered with strains from the Middle East, Europe, Japan, Pakistan, Bangladesh, and South Africa with 96.9–99.2% nucleotide similarity. P[8]-lineage 5 was exclusively formed of non-breakthrough specimens (*n* = 14) while lineage 6 included only one breakthrough isolate. In contrast to the VP7 gene, pairwise comparison revealed that the VP4 gene of P[8] breakthrough and non-breakthrough specimens shared slightly higher nucleotide and amino acid identities (88.4–93.5% and 90.6–95.9%, respectively) to the P[8] of RotaTeq® than that included in Rotarix® (88.4–91.2% and 90.0–94.8%, nucleotide and amino acid identities, respectively) (Table 2).

Lebanese P[4] RVA specimens were accommodated in three lineages with the majority (*n* = 15) clustering in P[4]-lineage 3. This lineage did not include any strains from other countries. P[4]-lineage 1 included six of the Lebanese RVAs along with strains from the USA, Australia, Africa, and South Asia with 98.5–99.0% nucleotide similarity. The remainder Lebanese P[4] RVAs (*n* = 12) co-clustered in lineage 2 with strains from Europe, Australia, Japan, and South Africa with 98.5–99.0% nucleotide similarity.

Aside from P[8]-lineage 5, no distinct clustering patterns were observed for Lebanese vaccine breakthrough specimens with the co-circulating non-vaccine breakthrough specimens.

We next compared the congruency in the tree topologies of the breakthrough and non-breakthrough specimens on the VP7 and VP4 phylogenetic trees. Generally, the VP4 and VP7 genes of the Lebanese RVAs belonged to similar clades in the respective gene trees, although some exceptions were noted in the VP4 gene segment. For instance, one G2 (NG184) and one G1 (N049) specimens clustered among G9 specimens in P[8]-lineage 4 comprised mainly of G9 specimens. This cluster also included a G1P[8] specimen from Belgium (BE2003). This tight clustering pattern suggests an intra-genotypic reassortment between G1P[8], G2P[8] and G9P[8] strains involving at least the VP4 gene. Similarly, G9 H017 and A095 specimens clustered tightly with G2 specimens in P[4] lineages 1 and 2, respectively, indicative of another potential reassortment event (Figure 2). P[4] lineage 1 also included another G9P[4] strain from the USA (LB1562). The detection of these potentially reassortant strains in other countries indicates that these strains were likely introduced to Lebanon rather than emerging locally.

### Changes in the VP7 Antigenic Epitopes of the Lebanese RVA Specimens Compared With the Vaccine Viruses

The VP7 protein plays a crucial role in stabilizing the viral outer capsid and is an important target for protective antibodies. Comparative studies of the VP7 protein indicated that substitutions occurring in two structurally defined antigenic epitopes [7-1 (7-1a and 7-1b) and 7-2], encompassing 29 amino acid residues, permit escape from neutralization by monoclonal antibodies (mAbs) (24, 25). mAbs targeting epitope 7-1 neutralize the virion through stabilizing the capsid and preventing uncoating, while the mechanism underlying the neutralization of epitope 7-2, the core of each VP7 subunit, is still not fully understood (25–27). Similarly, substitutions in these, as well as other, antigenic epitopes of the VP7 and VP4 may compromise the effectiveness of the Rotarix® and RotaTeq® vaccines (28, 29). Therefore, we assessed the presence of such substitutions by comparing the amino acid sequences of the VP7 and VP4 proteins of the Lebanese breakthrough and non-breakthrough RVA specimens with those of the vaccine strains.

**Figure 3:**
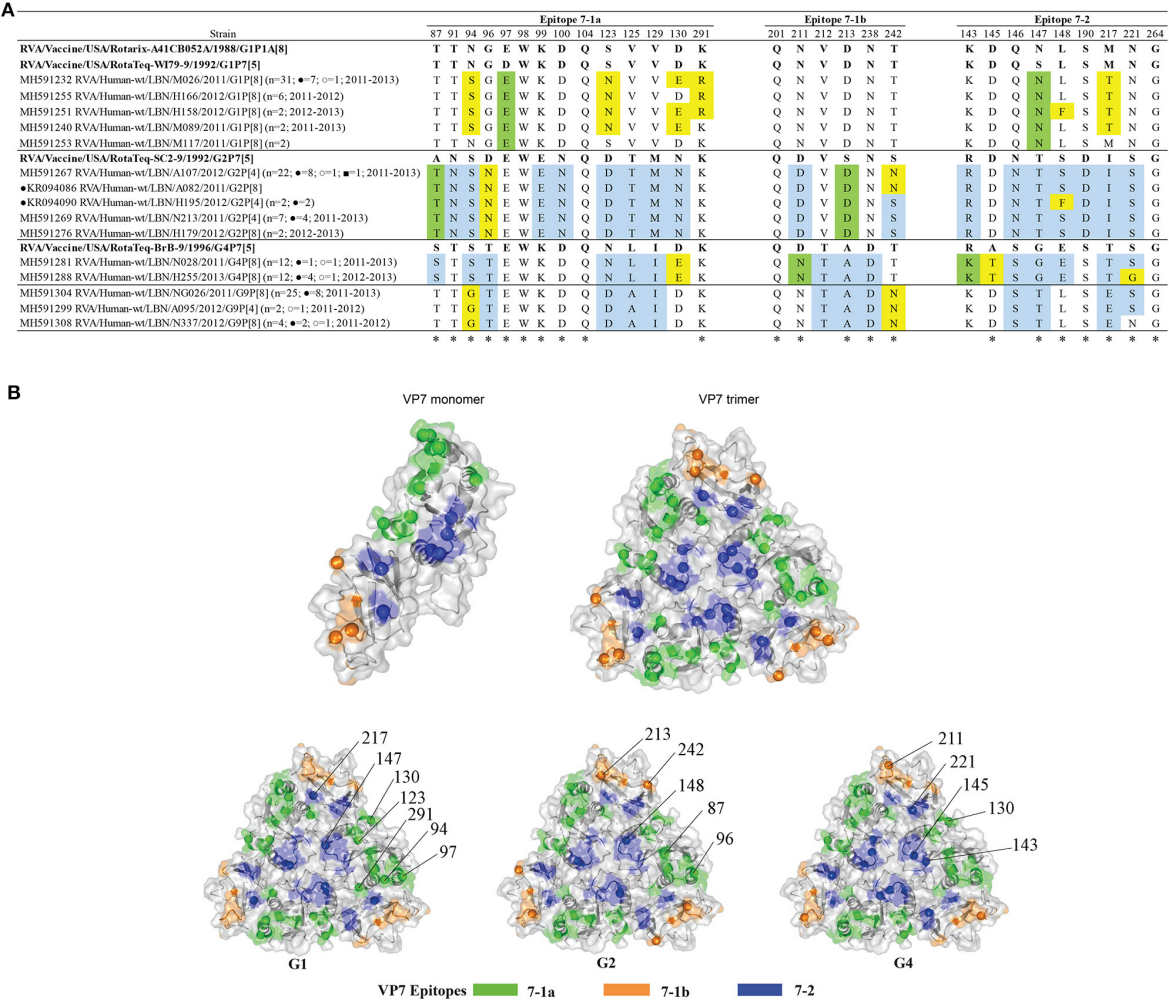
**(A)** Alignment of the antigenic epitopes of VP7 of the Lebanese RVAs with those of the vaccines. Representative Lebanese strains corresponding to those in the VP7 tree are shown. Residues that are different from Rotarix® and RotaTeq® are highlighted in yellow, in green-color are residues that are different from the respective genotypic strain in RotaTeq®, and in blue-color are residues that are different from Rotarix®. Amino acid residues known to mediate escape from neutralization with mAbs (2, 25) are indicated by an asterisk (*). **(B)** Surface representation cartoon of the VP7 monomer and trimer (PDB 3FMG). Antigenic epitopes are colored in green (7-1a), orange (7-1b), and blue (7–2). Substitutions relevant to parent vaccine strains are shown in spheres.

Alignment of the deduced amino acid sequences of the VP7 protein of the Lebanese breakthrough and non-breakthrough specimens with the vaccine strains showed that only four amino acids were fully conserved amongst all the detected genotypes (Figure 3A). The G1 breakthrough and non-breakthrough strains exhibited five substitutions when compared to the G1 strains of both Rotarix® and RotaTeq® strains. Four of these substitutions (N94S, S123N, D130E, and K291R) belonged to the 7-1a epitope, and one (M217T) occurred in the 7-2 epitope. Two G1 non-breakthrough specimens (N570 and H158) exhibited an additional substitution in epitope 7-2 (L148F). The G1 breakthrough and non-breakthrough specimens acquired two additional changes compared to G1 of RotaTeq®: D97E and S147N, occurring in the 7-1a and 7-2 epitopes. Mapping of these variations onto the VP7 trimer revealed that they tend to heterogeneously span the VP7 surface (Figure 3B). Interestingly, two of the non-breakthrough G1 isolates (M117 and A017) showed changes with respect to the RotaTeq®- G1 strain but not that of the Rotarix®.

All G2 breakthrough and non-breakthrough specimens possessed three substitutions in their VP7 antigenic epitopes when compared to G2 of RotaTeq®: A87T and D96N located in the 7-1a epitope and S213D in the 7-1b epitope. In addition, 23 of the 34 G2 specimens displayed an additional antigenic site substitution S242N in the 7-1b region; and two G2 breakthrough specimens possessed a unique change, S148F, in the 7-2 region (Figure 3A). The G2 substitutions tended to localize on the edges of the VP7 trimer (Figure 3B).

G4 breakthrough and non-breakthrough specimens possessed four substitutions in the VP7 epitopes when compared to G4 of RotaTeq®: D130E in the 7-1a, D211N in the 7-1b, and R143K and A145T in the 7-2 epitope. Half of the G4 specimens (*n* = 12) featured an additional S221G substitution in the 7-2 epitope region (Figure 3A). These substitutions were heterogeneously located across the surface of the VP7 trimer (Figure 3B).

All the G1 VP7 specimens possessed two conserved potential N-linked glycosylation sites at positions 69 and 238, which are also present in the vaccine strains. G2 VP7 possessed three potential N-linked glycosylation sites at positions 69-71, 146-148, and 238-240, which were found to be conserved in all except for the two G2 breakthrough specimens (due to an S148F substitution). These three N-glycosylation sites are conserved in the G2 of RotaTeq® vaccine. The VP7 of the G4 specimens possessed only the potential N-linked glycosylation site at position 69, which is also present in the G4 component of RotaTeq® vaccine. Noteworthy, all the Lebanese G4 strains exhibited an insertion of an asparagine (N) residue at position 76 adjacent to the mentioned N-glycosylation site in the hydrophilic region which was in congruency with global strains clustered in the same lineage. This substitution was also observed in all the G4 strains from other countries. Of note, eight Lebanese G9 RVA specimens, of which two are breakthroughs, possessed a unique alanine to valine substitution at position 68 (A68V) adjacent to the N-linked glycosylation site spanning residues 69-71, previously speculated to potentially alter the glycosylation and antigenicity of VP7 (30). Interestingly, this VP7 substitution was not observed in any of the global strains that clustered with G9 strains.

### Comparison of the VP4 Antigenic Epitopes of the Lebanese RVA Specimens and the Vaccine Viruses

The spike protein, VP4, mediates virion attachment and subsequent cell penetration. It also controls the host range and virulence of RV (31). Activation of RV requires the proteolytic cleavage of VP4 by the action of intestinal trypsin into N- and C-terminal polypeptide fragments, VP8^*^ and VP5^*^, respectively (32). The outer capsid protein VP5^*^, which forms the spike's body, acts as a membrane penetration protein that mediates virion release from ER to the cytoplasm (33). The VP8^*^ forms the globular head of the spike and mediates host cell attachment (34). VP8^*^ and VP5^*^ contain four (8-1 to 8-4) and five (5-1 to 5-5) epitopes, respectively, which are targets for neutralizing mAbs. Neutralizing antibodies directed against the 5-1 epitope region seem to block virion membrane penetration (27), and those targeting the VP8^*^ neutralize virus infectivity by inhibiting viral attachment (35).

**Figure 4:**
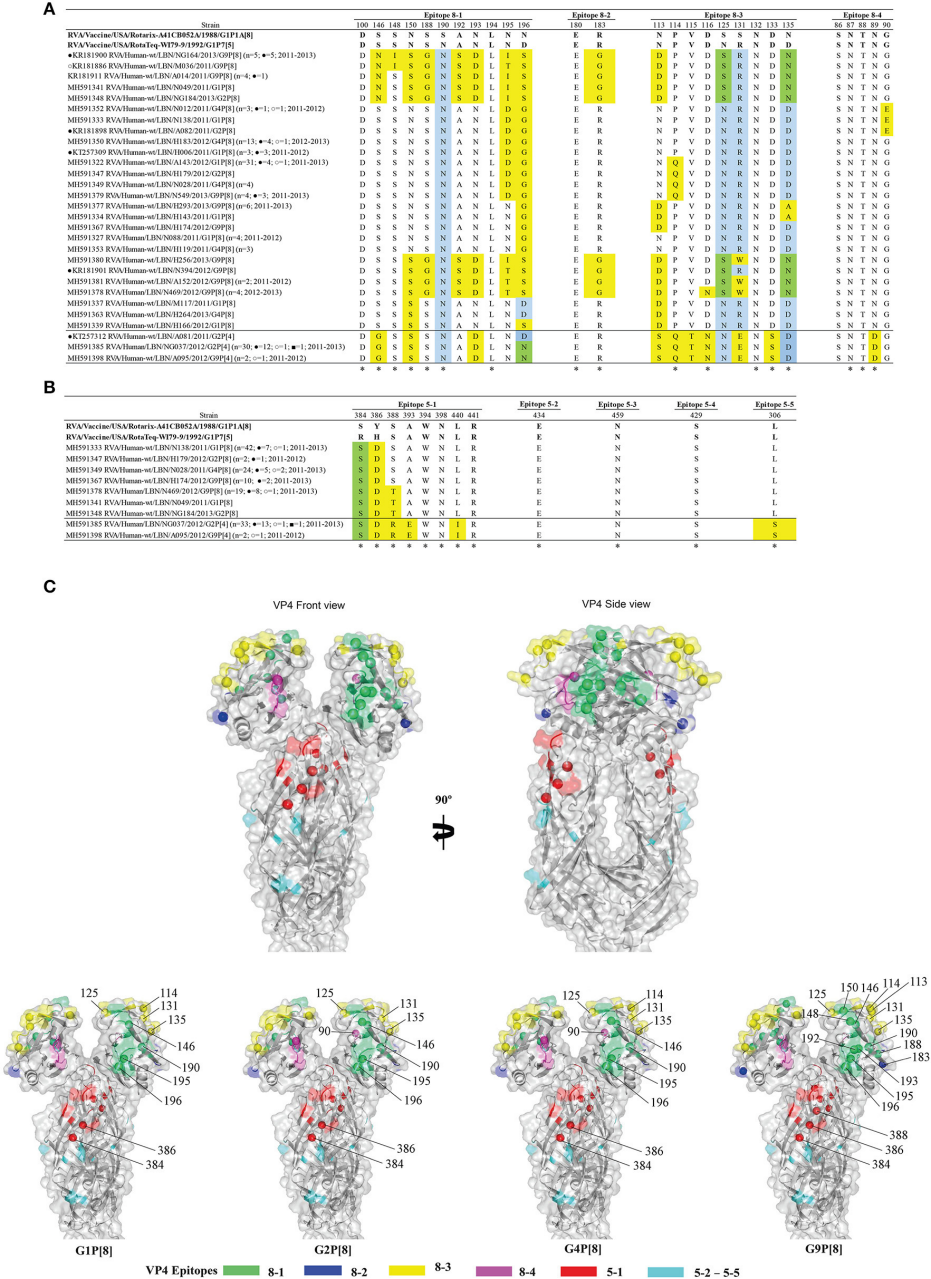
Alignment of the antigenic epitopes of VP4 of the Lebanese RVAs with those of Rotarix® and RotaTeq®. Representative Lebanese strains corresponding to those in the VP4 tree are shown. **(A)** Alignment of the antigenic epitopes of the VP8 of the vaccines and representative RVAs circulating in Lebanon. **(B)** Alignment of the antigenic epitopes of the VP5 of the vaccines and representative RVAs circulating in Lebanon. Residues that are marked by yellow-color are residues that are different from Rotarix® and RotaTeq®, in green-color are residues that are different from the respective genotypic strain in RotaTeq®, and blue-color are the residues that are different from Rotarix®. Amino acid residues known to mediate escape from neutralization with mAbs (2) are indicated by an asterisk (*). **(C)** Surface representation cartoon of the VP4 protein (PDB 3GZT). The right image is rotated 90° compared to the left image. Antigenic epitopes are colored in green (8–1), blue (8–2), yellow (8–3), pink (8–4), red (5–1), and cyan (5-2 to 5-5). Substitutions relevant to parent vaccine strains are shown in spheres.

Alignment of the deduced amino acid sequences of the VP4 proteins amongst all specimens with the vaccine strains revealed 14 fully conserved amino acid residues (Figures 4A,B). Among the P[8] specimens, the VP4 of G9 specimens possessed the highest variability compared to P[8] of Rotarix® and RotaTeq®. These viruses had 16-17 and 16-18 substitutions in their VP4 antigenic epitopes compared with the P[8] of Rotarix® and RotaTeq®, respectively. These specimens clustered with the P[8] OP354-like RVA lineage (lineage 4, represented by Belgium BE1418 G9P[8] strain); a genetically distinct and rarely reported lineage (36). Overall, the majority of substitutions observed in the Lebanese RVA viruses were located in the globular head (VP8^*^) of the VP7 protein and very few were mapped to the spike's body or VP5^*^ (Figure 4C).

Moreover, all Lebanese P[4] specimens, except for one (A081) shared a similar VP4 antigenic residues profile compared to the P[8] strains of the vaccines. The breakthrough G2P[4] specimen (A081) possessed an aspartic acid (D) at position 196, similar to RotaTeq®, while the remainder Lebanese specimens had an asparagine (N) like Rotarix® (Figures 4A,B).

## Discussion

Currently, two RV vaccines Rotarix® and RotaTeq® are available worldwide for the prevention of RV infections with proven safety profiles (9, 10, 12, 37). Both vaccines are highly efficacious against the commonly circulating RV genotypes including G1-4, G9, and P[4], [6], and [8] (12). Accordingly, the WHO recommended the implementation of RV vaccination in the NIPs to curb the worldwide health burden due to RV morbidity and mortality (2, 8). RV vaccines have demonstrated higher efficacy in developed countries (85%) compared to developing countries (61%) (37). Several post-licensure studies have shown a decrease in RV infections and associated hospitalizations in developing countries upon the implementation of RV vaccination (17).

Vaccine use can impose a selective pressure on circulating strains, which could result in a shift toward otherwise less common genotypes or selection of mutant viruses that are not effectively neutralized. For instance, the introduction of Rotarix® in Belgium was associated with a decrease in the vaccine homotypic strains (38). Similar observations were also reported in other developing and developed countries (37, 39). In countries with high coverage of RotaTeq®, increases in the prevalence of uncommon and unusual strains were reported (40, 41).

In Lebanon, where the majority of infants receive Rotarix®, G1P[8] was the dominant genotype post-vaccine introduction compared to G4P[8] in the years before vaccine introduction (16, 42). In fact, the G1 VP7 and P[8] VP4 genes of the breakthrough and non-breakthrough strains were more similar to Rotarix® than RotaTeq®, despite belonging to non-vaccine G1 lineages. This RVA genotype pattern in Lebanon can be explained by the low vaccination coverage in Lebanon that does not support population-level selective pressure on circulating strains. Even in the Eastern Mediterranean Region (EMR) countries where RVA vaccines have been introduced to their NIPs, vaccination coverage is <30% excluding the possibility of selective pressure at the regional population level (43). While high-income countries in the EMR have vaccination coverage of 82% (43), the regional trends in vaccine usage might eclipse the effect of local vaccination practices. In Saudi Arabia, where vaccine coverage increased from 84% in 2013 to 94% in 2014, G1P[8] was still the predominantly circulating genotype in 2015 (44). Similar findings were also reported in Morocco with high vaccine coverage (45).

Serotype-specific immunity induced by both RV vaccines against circulating G1P[8] RVAs has been, by far, the most commonly established mechanism of protection. A recent study conducted by Donato et al., from Northern Australia described a G1P[8] outbreak including vaccinated children, 43 months following Rotarix® implementation (46). The Australian outbreak strain had several variations in its VP7 trimeric protein, namely: T91N, S123N, N94S, M217T, and K291R; and Y385D in VP5^*^; all of which correlated with neutralization-resistance against a panel of mAbs and polyclonal sera (46). S123N, N94S, and M217T variants were detected among all, except two non-breakthrough (M117 and A017), Lebanese G1 VP7 isolates. Additionally, the K291R variant was detected among all G1 VP7 specimens, except four non-breakthrough strains (M117, A017, M089, and N525). Therefore, the detection of these substitutions among circulating RVAs in Lebanon may have implications on the effectiveness of vaccines. Also, the detection of these variants in other countries indicates that they were imported to Lebanon rather than having emerged locally under selective vaccine pressure. This is supported by a high genetic homology of VP7 and VP4 genes of the Australian outbreak G1P[8] with strains circulating in Asia and Belgium (46).

Considering RVA frequency by VP7 and VP4 combination, G2P[4] was the most frequently detected RVA breakthrough genotype in Lebanon (16). A higher incidence of G2P[4] strains was previously reported in several countries after implementing Rotarix® in the NIPs as the main or only vaccine (38, 39, 47, 48). Given the high mutation rate (1.45 × 10^−3^ mutations/site/year) of the G2 VP7 gene, G2 strains have been rapidly evolving and spreading during the last decade (49, 50). Globally circulating G2P[4] strains were reported to belong to four lineages (I-IV) as compared to the prototypic DS-1 (G2) human strain (49), with lineage IV dominating since the early 2000s. The G2 IVa sublineage features a D96N substitution in the VP7 7-1a epitope, also detected among Lebanese G2 RVAs, that was associated with the massive increase of G2P[4] during the last decade (49–52). More recently, this lineage selected an S242N in the VP7 gene, similar to some of our G2 specimens, and has been sporadically reported worldwide. Detection of both variants in Lebanon, during and after the 2010-2011 RV seasons, suggests that these substitutions were conserved and might confer a fitness advantage for the G2 viruses.

The VP4 gene is more likely to be influenced by negative selection due to the important structural and functional roles it plays, including attachment, penetration, and maturation. Therefore, it is less diverse, compared to the VP7 (27, 53). In concordance, 30.8% (12/39) of the residues belonging to the antigenic epitopes of the VP4 of the Lebanese RVA specimens were fully conserved compared to the vaccine strains vs. only 13.8% (4/29) in the VP7. Therefore, while the high variability in the VP7 genes could undermine vaccine-induced immunity, the VP4 component of the vaccine strains might be able to compensate for the weakened neutralizing capacity of vaccine-induced antibodies when a patient is infected with a P[8] strain. However, when the VP4 genotype of the infecting strain is non-P[8], then the protection afforded by the vaccines will mainly depend on the VP7 component and might thus fail in case of substitutions in the antigenic epitopes.

Glycosylation of the RV glycoproteins modifies their antigenicity and promotes their resistance to neutralizing mAbs (54). Gain or loss of glycosylated residues was detected in RV antigenic variants that are resistant to mAbs (54, 55). Such changes were also predicted to correlate with the virulence of RV strains (56). The G1 VP7 of the vaccine strains possessed two predicted N-linked glycosylation sites at positions 69 and 238, which were conserved in the Lebanese RVA specimens. Similarly, all Lebanese RVA strains, except two G2 breakthrough specimens (H195 & H198) maintained two out of three potentially N-glycosylated residues present in the corresponding RotaTeq® vaccine component. These two specimens possessed an S148F substitution, which results in a loss of the potential glycosylation at site 146-148. Changes at this residue have been previously shown to induce resistance to neutralizing antibodies targeting the 7-2 epitope of the VP7 (55). As such, a loss of glycosylation on this residue is likely to have altered the antigenicity of these breakthrough strains. In the case of the Lebanese G4 RVAs, all of the specimens maintained the N-glycosylated residue at site 69-71 present in the corresponding vaccine strain. However, insertion of an asparagine detected among the Lebanese G4 strains at residue 76 neighboring this glycosylated site might affect the conformation of the VP7 protein and alter its antigenicity. This insertion was previously reported among the G4P[8] RV strains in Argentina, Uruguay, Brazil, and Nicaragua during 1998 to 2005 and was found, along with other substitutions in the region, to structurally modify the two downstream β-sheets at amino acid positions 80 to 85 and 115 to 119 (57). Another substitution that could have altered the antigenicity of the Lebanese RVA strains was detected among all G9 specimens at residue 68 close to the glycosylation site at amino acids 69-71, which lie within a predicted immunodominant domain (30). Monitoring the conservation and spread of these substitution over time will provide insight into their effect on viral fitness.

A new strain of RVA, known as OP354-like, possessing a genetically divergent P[8] VP4 has recently emerged worldwide (36). Zeller et al. suggested that this strain has emerged in South and East Asia and spread to other regions in Africa, Europe, and North America between 1998 and 2012 (36). OP354-like P[8] isolates were genetically distant from all other P[8] lineages, including those containing the Rotarix® and RotaTeq® P[8] VP4 genes, which could lead to reduced vaccine effectiveness. Our analysis revealed that this lineage was imported to Lebanon. OP354-like P[8] genotype was detected among 20 Lebanese RVA strains of G9 (18/20), G1 (1/20), and G2 (1/20) genotype. Notably, these strains displayed the highest genetic diversity and the largest number of antigenic substitution compared to the rest of P[8] surveyed RVAs. These changes might be partially responsible for the fitness advantage that allowed this lineage to spread globally during a relatively short period and to escape neutralization induced by the available vaccines.

This is the first report on RVA genetic diversity in Lebanon. RVAs circulating in Lebanon including breakthrough strains are diverse and possess many substitutions in the antigenic epitopes that were previously associated with escape from neutralizing mAbs. However, a limitation of this study was the lack of serum specimens from vaccinated and non-vaccinated patients to assess the immunologic status of these patients and explain the potential cause of breakthrough infections. The Lebanese strains were similar to RVAs circulating in other countries. We also report for the first time the spread of the recently emerging and rare OP354-like P[8] RV genotype to the Middle East, including Lebanon. Continuous monitoring of circulating RVAs in parallel with the assessment of their vaccine matching using genotype-specific antibodies and neutralization assays are warranted to maintain the effectiveness of current vaccines.

## Data Availability Statement

The datasets generated for this study can be found in the GenBank.

## Ethics Statement

The studies involving human participants were reviewed and approved by the Institutional Review Board, American University of Beirut. Written informed consent to participate in this study was provided by the participants' legal guardian/next of kin.

## Author Contributions

HZ, GD, and GM conceived and designed the study. HH, LR, and AS conducted the experiments. ZA and MH collected the samples. HH and HZ analyzed the data and wrote the manuscript. All authors revised and approved the final draft.

### Conflict of Interest

The authors declare that the research was conducted in the absence of any commercial or financial relationships that could be construed as a potential conflict of interest.
